# West Nile and St. Louis encephalitis viral genetic determinants of avian host competence

**DOI:** 10.1371/journal.pntd.0006302

**Published:** 2018-02-15

**Authors:** Payal D. Maharaj, Angela M. Bosco-Lauth, Stanley A. Langevin, Michael Anishchenko, Richard A. Bowen, William K. Reisen, Aaron C. Brault

**Affiliations:** 1 Division of Vector-Borne Diseases, Arboviral Disease Branch, Centers for Disease Control and Prevention, Fort Collins, Colorado, United States of America; 2 Center for Vectorborne Disease Research and Department of Pathology, Microbiology and Immunology, School of Veterinary Medicine, University of California, Davis, California, United States of America; 3 Department of Biomedical Sciences, Colorado State University, Fort Collins, Colorado, United States of America; Oregon Health and Science University, UNITED STATES

## Abstract

West Nile virus (WNV) and St. Louis encephalitis (SLEV) virus are enzootically maintained in North America in cycles involving the same mosquito vectors and similar avian hosts. However, these viruses exhibit dissimilar viremia and virulence phenotypes in birds: WNV is associated with high magnitude viremias that can result in mortality in certain species such as American crows (AMCRs, *Corvus brachyrhynchos*) whereas SLEV infection yields lower viremias that have not been associated with avian mortality. Cross-neutralization of these viruses in avian sera has been proposed to explain the reduced circulation of SLEV since the introduction of WNV in North America; however, in 2015, both viruses were the etiologic agents of concurrent human encephalitis outbreaks in Arizona, indicating the need to re-evaluate host factors and cross-neutralization responses as factors potentially affecting viral co-circulation. Reciprocal chimeric WNV and SLEV viruses were constructed by interchanging the pre-membrane (prM)-envelope (E) genes, and viruses subsequently generated were utilized herein for the inoculation of three different avian species: house sparrows (HOSPs; *Passer domesticus*), house finches (*Haemorhous mexicanus*) and AMCRs. Cross-protective immunity between parental and chimeric viruses were also assessed in HOSPs. Results indicated that the prM-E genes did not modulate avian replication or virulence differences between WNV and SLEV in any of the three avian species. However, WNV-prME proteins did dictate cross-protective immunity between these antigenically heterologous viruses. Our data provides further evidence of the important role that the WNV / SLEV viral non-structural genetic elements play in viral replication, avian host competence and virulence.

## Author summary

Since the identification of West Nile virus (WNV) in North America in 1999, St. Louis encephalitis virus (SLEV) cases declined rapidly. Both viruses utilize similar avian hosts and vectors for maintenance of transmission cycles; however, they present different phenotypes in both vector and avian host. In birds, WNV develops high viremias and elicits mortality whereas SLEV has not been associated with avian virulence. West Nile viral non-structural genetic elements have been demonstrated herein to dictate higher viremias in competent avian hosts and virulence in AMCRs. In contrast, non-structural SLEV elements previously have been shown to dictate increased oral infectivity in *Culex* mosquitoes, likely as a compensation for the lower viremias generated by SLEV. These findings coupled with the co-circulation of WNV and SLEV in Arizona in 2015 demonstrate that pre-existing flaviviral immunity does not necessarily preclude concurrent circulation of these viruses.

## Introduction

West Nile (WNV) and St. Louis encephalitis (SLEV) viruses are serologically related flaviviruses (*Flavivirus*; *Flaviviridae*) that enzootically circulate between *Culex* spp. mosquitoes and avian amplification hosts in North America with the potential to give rise to epidemics of human encephalitis [1]. West Nile virus is capable of eliciting extended, high-magnitude viremias and mortality in a wide range of avian species including house sparrows (HOSPs, *Passer domesticus*), house finches (HOFIs, *Haemorhous mexicanus*) and American crows (AMCRs, *Corvus brachyrhynchos*) [2, 3]. Additionally, HOSPs and HOFIs are both competent host species for SLEV in North America [4–6]; however, WNV and SLEV exhibit differential virulence and mortality phenotypes in these species [7, 8]. American crows are highly susceptible to WNV infection and can generate serum viremias in excess of 11 log_10_ PFU/mL sera [2]. Consequently, they have been utilized as a sentinel species to track the spread of the virus in North America [9]. In contrast, SLEV presents low-level viremias in a more restricted range of birds and has not been associated with mortality in adult birds [1]. Since its first identification in North America in 1999, WNV has expanded its geographic range and established enzootic transmission foci throughout the continent [10]. Concurrent with this introduction and subsequent geographic radiation of WNV, SLEV enzootic activity has waned despite the increased levels of arboviral surveillance precipitated by the WNV invasion [11].

Several studies have assessed the neutralizing cross-protective effect of immune responses to heterologous flaviviruses [7, 12–14]. Given these previous findings and the overlapping distributions of WNV and SLEV in the New World, it is apparent that improved understanding of the cross-protective immune responses in avian hosts between sequential WNV and SLEV infections will be imperative to gain a complete understanding of flaviviral transmission dynamics in North America [15–18]. Reports have shown that prior infection of HOFIs with WNV completely ablated SLEV viremias following secondary challenge, whereas primary infection with SLEV retarded WNV viral titers by 1,000-fold, leading to a reduction in the length of acute viremia and the prevention of mortality [16]. Concurrent outbreaks of SLEV and WNV human encephalitis was documented in Arizona in 2015 [18] during the same time period that SLEV was isolated in southeastern California concurrently with WNV, representing the first isolation of SLEV in California in more than twelve years [19].

A wide variety of factors dictate the efficiency of flavivirus transmission including: the relative susceptibility of mosquito vectors to oral infection and avian host competence related to herd immune status, viral genetic elements that dictate oral vector susceptibility and avian viremias, and environmental conditions. Mapping of viral genetic determinants of flavivirus host virulence through utilization of chimeric viral constructs has been described extensively for other flaviviruses such as tick-borne encephalitis (TBEV) and yellow fever (YFV) viruses [20–22]. Various studies have provided conclusive evidence of specific genetic elements that influence WNV host virulence phenotypes such as plaque morphology, murine neuroinvasiveness, temperature sensitivity and avian viremia profiles in AMCRs [23–25]. Prior studies have demonstrated that WNV non-structural genetic elements can dictate AMCR virulence phenotypes [2, 25, 26]. However, genetic differences between these viruses have not been previously studied to elucidate the viral determinant(s) dictating the avian host phenotype differences exhibited by WNV and SLEV. Structural chimeric viruses of WNV and SLEV, WNV-prME/SLEV.IC and SLEV-prME/WNV.IC, were previously used to demonstrate that non-structural genetic elements of WNV dictated increased *in vitro* growth capacity in avian cell culture [27].

To assess whether the high avian *in vitro* replication phenotype of WNV associated with non-structural genetic determinants could be recapitulated *in vivo*, host competence studies were conducted in HOSP, HOFI and AMCRs. Furthermore, to identify specific viral genetic determinants of cross-protective immunity in passerine hosts, previously infected HOSPs were challenged with antigenically heterologous WNV or SLEV viruses to characterize immune responses and viremia profiles following secondary challenge.

## Methods

### Cells and viruses

African green monkey kidney cells (Vero) were maintained at 37°C with 5% CO_2_ in Dulbecco’s Modified Eagle Medium (DMEM; Gibco, Carlsbad, CA) supplemented with 10% fetal bovine serum (FBS) and penicillin/streptomycin (100U/mL and 100μg/mL, respectively). The parental SLEV strain IMP115 was isolated in 2003 from a pool of *Cx*. *tarsalis* mosquitoes from Imperial Valley, CA and was passaged once in Vero cells [27]. Infectious cDNA clone-derived viruses of WNV NY99 strain (WNV.IC), SLEV IMP 115 [28, 29] and chimeric viruses, SLEV-prME/WNV.IC and WNV-prME/SLEV.IC [28, 29], were rescued from full-length infectious cDNAs as described previously [27]. All viruses were passaged once in Vero cells. WNV.IC and SLEV-prME/WNV.IC viruses were harvested at 3 days post-infection (dpi) and SLEV/IMP115, SLEV.IC and WNV-prME/SLEV.IC were harvested at 7 dpi. The complete genomes of the rescued viruses were sequenced to confirm the genetic identity of all parental and chimeric viruses utilized for subsequent experiments as described previously [27, 28].

### Bird collection

House sparrows and HOFIs were collected in ground traps in vineyards near Bakersfield, Kern County, California in late summer of 2007 (Table 1). Additional HOSPs were captured in Larimer County, Colorado using mist-nets and/or ground grain-baited traps in late summer of 2010 (Table 2) and AMCRs were collected in Oklahoma in 2011 using cannon nets (Table 1). Birds were banded, bled to determine previous infection status, and then housed in groups of 5–6 per cage within a containment laboratory for two weeks to ensure cage adaptation and general health. Wild birdseed mix and fresh water were provided *ad libitum* with sand being supplied as a digestive supplement for HOSP and HOFI. Crows were provided a mixture of cat and dog food *ad libitum*.

**Table 1:** Summary of infection studies performed in house finches, house sparrows and American crows.

House finches (2007)	Sample size	House sparrows (2007)	Sample size	American crows (2011)	Sample size
WNV.IC	5	WNV.IC	6	WNV.IC	5
SLEV-prME/WNV.IC	5	SLEV-prME/WNV.IC	6	SLEV-prME/WNV.IC	5
SLEV/IMP115	6	SLEV/IMP115	6	WNV-prME/SLEV.IC	5
-	-	-	-	SLEV.IC	5

**Table 2:** Summary of infection and challenge study performed in house sparrows from Colorado (2010).

Group	Infection Study in house sparrows			Challenge Study performed in previously infected house sparrows*		
	Initial infection	Sample size	Immune Status after infection	Challenge infection	Sample size**	Immune status after challenge
1	SLEV/IMP115	8	SLEV/IMP115 immune	WNV.IC	8	SLEV/IMP115 immune: WNV.IC challenge
2	SLEV.IC	8	SLEV.IC immune	WNV-prME/SLEV.IC	7	SLEV.IC immune: WNV-prME/SLEV.IC challenge
3	SLEV-prME/WNV.IC	8	SLEV-prME/WNV.IC immune	WNV-prME/SLEV.IC	5	SLEV-prME/WNV.IC immune: WNV-prME/SLEV.IC challenge
4	WNV-prME/SLEV.IC	7	WNV-prME/SLEV.IC immune	SLEV-prME/WNV.IC	7	WNV-prME/SLEV.IC immune: SLEV-prME/SLEV.IC challenge
5	WNV.IC	8	WNV.IC immune	SLEV-prME/WNV.IC	6	WNV.IC immune: SLEV-prME/WNV.IC challenge

*Three weeks after the infection study, the same groups of house sparrows were challenged with antigenically heterologous flaviviruses.

** Samples sizes were lower due to some mortality during the initial infection period

### Serology

Serum samples were taken from all birds used for experimentation and were tested for WNV and/or SLEV reactive and neutralizing antibodies by enzyme immunoassay (EIA) as described previously [30] and plaque reduction neutralization tests, respectively, at the time of capture. From Californian HOSP and HOFIs, 0.1mL samples of whole blood were drawn and diluted in 0.9mL of PBS, clarified by centrifugation, frozen at -80°C, heat-inactivated at 56°C for 30 minutes and then assessed for antibodies against WNV or SLEV using an immunoassay described previously [30]. A 0.1mL sample of blood was drawn from HOSPs from Colorado and 0.2mL was drawn from AMCRs collected in Oklahoma by jugular venipuncture and used in a standard plaque reduction-neutralization assay (PRNT_90_) as previously described [31]. Briefly, blood drawn from each bird was placed in serum separator tubes (Becton Dickinson, Franklin Lakes, NJ) and sera separated through centrifugation at 5,000 x g and subsequently stored at -20°C. Serum samples were heat-inactivated at 56°C for 30 minutes, diluted 1:10 and serially diluted 2-fold in 96-well plates. The reciprocal of the serum dilution yielding a ≥90% reduction in the number of plaques (PRNT_90_) compared to a serum negative control was used as the threshold for assessing a neutralization response.

### Infection study

Preliminary inoculation studies included in this report were performed with California HOSPs and HOFIs prior to the generation of the SLEV.IC and WNV-prME/SLEV.IC infectious clone derived viruses (Table 1) in 2007. In the California HOFI study, a WNV seropositive HOFI was inadvertently inoculated with SLEV-prME/WNV.IC as the HOFI was EIA and plaque negative at the time of capture, but had seroconverted prior to the time of inoculation. Blood samples were collected from this HOFI as well. All subsequent avian experimental infection studies were performed in Colorado during and after 2010 and utilized the rescued SLEV and WNV parental and chimeric infectious clone viruses described previously [27]. Flavivirus seronegative HOSPs, HOFIs and AMCRs were needle- inoculated subcutaneously in the cervical region with 1,000–1,500 PFU/ 0.1mL of virus diluted in phosphate buffered saline (PBS) (Tables 1 and 2). To ensure phenotypic concordance of the SLEV.IC with its parental virus, SLEV/IMP115, CO HOSPs were also inoculated with the field isolated SLEV/IMP115 virus. Birds were monitored daily for clinical signs of disease such as lethargy, fluffed feathers, decreased activity and emaciation [13, 32]. A 0.1mL aliquot of blood was drawn daily from HOSPs and HOFIs from the jugular vein from 1 to 7 dpi, diluted in 0.4mL of BA-1 media supplemented with 20% FBS, allowed to coagulate and centrifuged to pellet clotted cells. Serum sampling from AMCRs was performed in an identical manner with the exception that 0.2mL of blood was added to 0.8mL BA-1 media. Samples were stored at -80°C until titrated for infectious units by plaque assay on Vero cells [31]. The limit of virus detection was 1.7 log_10_ PFU/mL of sera based on a 0.2 mL plaque assay inocula per well of the initial 1:10 diluted sera samples. Daily mean titers were calculated and plotted as a line graph against days post inoculation. Mean peak titers for each group were calculated by averaging the peak titer of each individual bird regardless of the day post-inoculation and were presented as scatter plots.

### Challenge study

At 21 dpi, surviving house sparrows from the infection study in which the birds were initially inoculated with SLEV/IMP 115, SLEV.IC, SLEV-prME/WNV.IC, WNV-prME/SLEV.IC or WNV.IC were challenged by needle inoculation with antigenically heterologous viruses, WNV.IC, WNV-prME/SLEV.IC or SLEV-prME/WNV.IC (Table 2). Blood was collected daily and titrated for infectious units by plaque assay as before. Scatter plots of daily virus titers for each individual challenged HOSP were plotted against days post-inoculation. The scatter plots were then compared with the daily mean viremia profiles of the same virus (with 95% confidence limits) generated in naïve HOSPs from the infection study (line graphs). Individual titers from the challenge infections that fell outside of the upper 95% confidence interval were considered significant.

### Immune responses of HOSPs to viral inoculation

To assess the relative magnitude of the homologous and heterologous flaviviral immune response induced in avian hosts following inoculation with the parental or chimeric viruses, PRNT_90_ titers against WNV.IC and the SLEV-prME/WNV.IC were determined for serum samples taken from birds 21 dpi following primary inoculation (Table 2). Previous studies demonstrated that SLEV-prME/WNV.IC was antigenically indistinguishable to SLEV.IC [27]. At 21 days post-secondary challenge, surviving HOSPs were terminally bled for sera to be used to assess neutralizing titers against WNV or SLEV-prME/WNV. A standard PRNT_90_ was performed using WNV.IC and SLEV-prME/WNV.IC to assess the presence and magnitude of homologous and heterologous neutralizing antibodies. The SLEV-prME/WNV.IC virus was used in place of SLEV as plaques could be visualized at 3 dpi while maintaining antigenic specificity for SLEV [27]. All HOSPs demonstrated a low level of non-specific neutralization at 1:10 serum dilutions, although this generally did not meet the 90% threshold used for the neutralization tests. Nevertheless, given this factor and the limited serum obtained from the birds, only 1:20 serum dilutions were used for screening for prior exposure to either WNV or SLEV as well as for PRNT_90_ determinations following initial and secondary inoculations.

### Statistical analysis

A repeated measures ANOVA was used to compare daily serum viremias over time among viruses, with mean viremias grouped by *a posteriori* Tukey-Kramer multiple comparison tests with α = 0.05 [33]. Peak mean titers were compared by a two-tailed student’s T-test with α = 0.05. In the challenge study, statistical significance was established by calculating 95% confidence limits (CIs) for daily mean titers for naïve HOSPs and comparing these with daily individual titers of secondarily challenged HOSPs (Table 2). Individual secondary challenge virus titers falling outside of the 95% CI were treated as significant.

### Ethics

The collection, housing, transport and inoculation of California and Colorado birds were conducted under approved University of California, Davis, Institutional Animal Care and Use Committee (IACUC) protocols 12876 and 12880 and Colorado State University, Institutional Animal Care and Use Committee protocol 10-2078A, respectively. Birds were collected by grain-baited traps and mist nets under USGS Master Station Banding Permit 22763 and State of California Scientific Collecting Permits, and taken for experimentation under Federal Permit MB082812. Laboratory facilities in Bakersfield were approved under BUA 0554 by the University of California, Davis, Environmental Health and Safety Committee, and USDA Permit 47901. Compliance of animal housing guidelines were followed as per requirements of IACUC 10-2078A in the Animal Disease Lab at Colorado State University. No endangered species were caught when trapping HOSPs, HOFI or AMCRs. All protocols and practices for the handling and manipulation of birds were in accordance with the guidelines of the American Veterinary Medical Association (AVMA) for humane treatment of laboratory animals as well as the ‘‘Guidelines to the Use of Wild Birds in Research” published by the ornithological council 3rd edition (2010).

## Results

### Differential virulence of West Nile virus and St. Louis encephalitis virus in house finches, house sparrows and American crows

**Fig 1:**
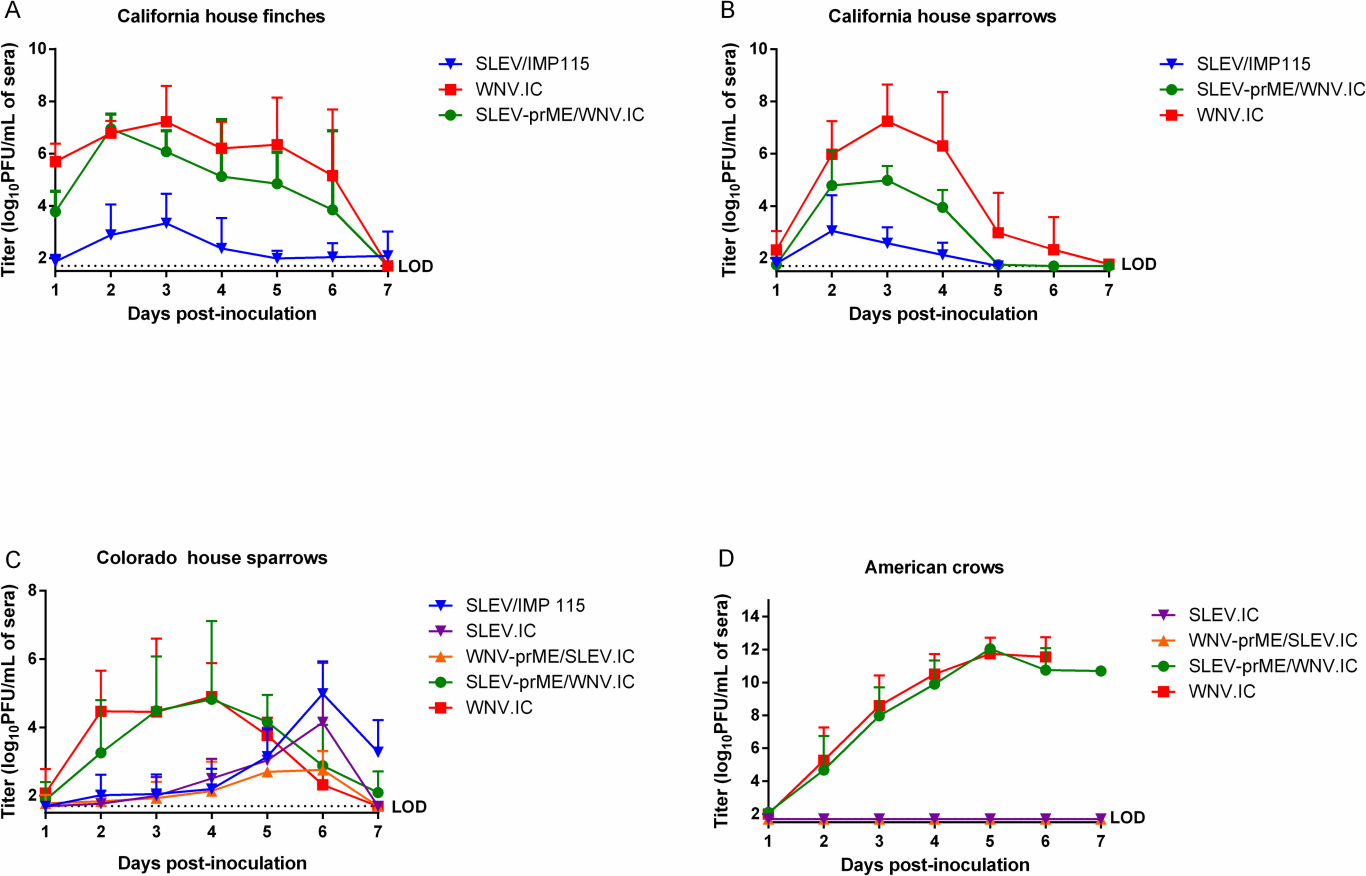
Daily mean virus serum titers (log_10_ PFU/mL + standard deviation) in different avian species each inoculated with 1500 PFU of SLEV-prME/WNV.IC, SLEV/IMP115, SLEV.IC, WNV-prME/SLEV.IC and WNV.IC viruses were assessed over 1–7 days post-inoculation: (A) California house finches, (B) California house sparrows, (C) Colorado house sparrows, (D) American crows. The detection limit (LOD) for this assay was 1.7 log_10_ PFU/mL.

**Fig 2:**
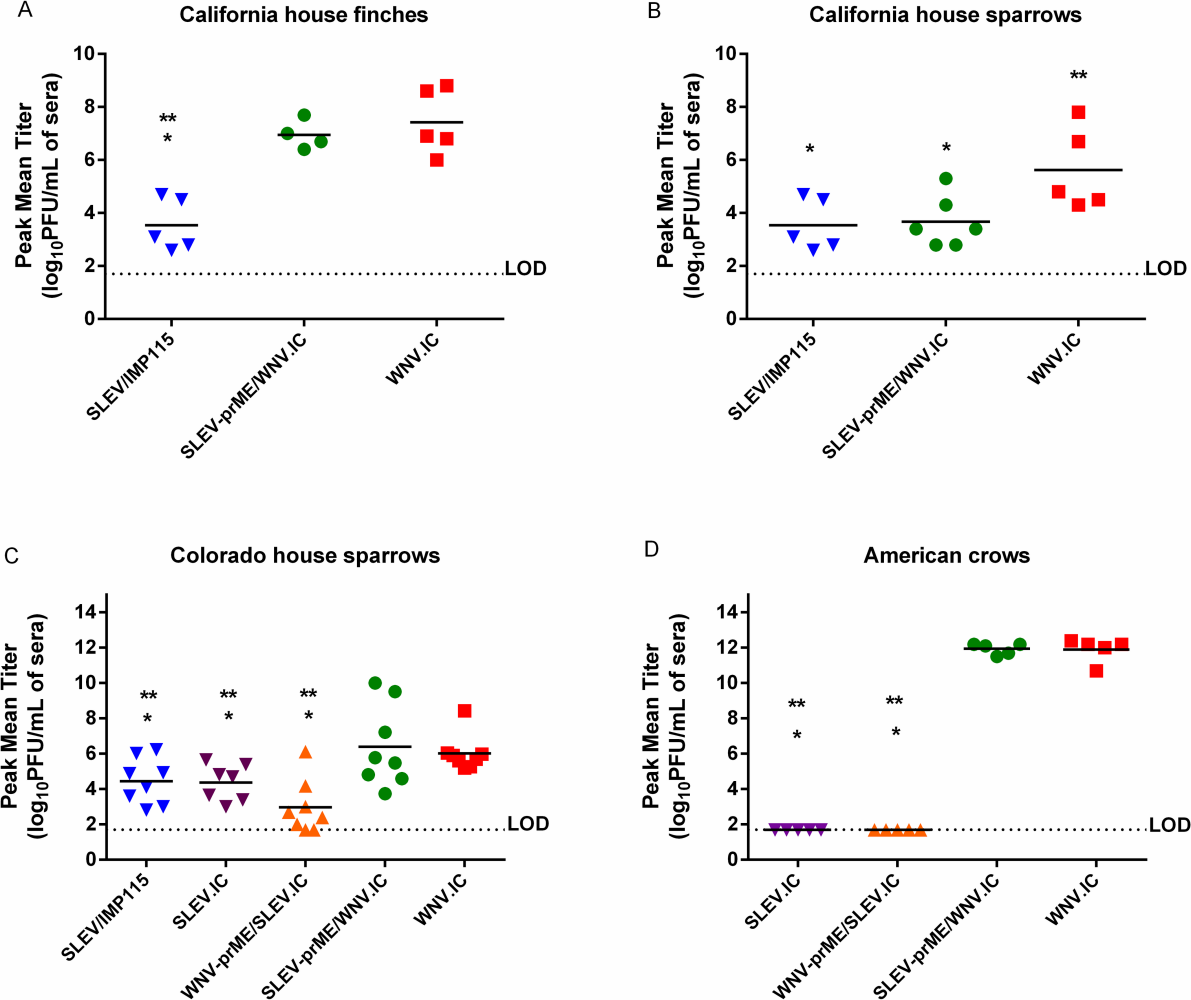
Peak mean virus serum titers (log_10_ PFU/mL) of individual birds inoculated with 1500 PFU each of SLEV-prME/WNV.IC, SLEV/IMP115, SLEV.IC, WNV-prME/SLEV.IC and WNV.IC: (A) California house finches, (B) California house sparrows, (C) Colorado house sparrows, (D) American crows. The detection limit for serum viremia titers was 1.7 log_10_ PFU/mL. Student’s T-tests were used to compare peak mean titers. Asterisks (*) represent statistical significance of p<0.05 when compared with WNV.IC and ** represent comparisons with SLEV-prME/WNV.IC at a p value of <0.05.

**Fig 3:**
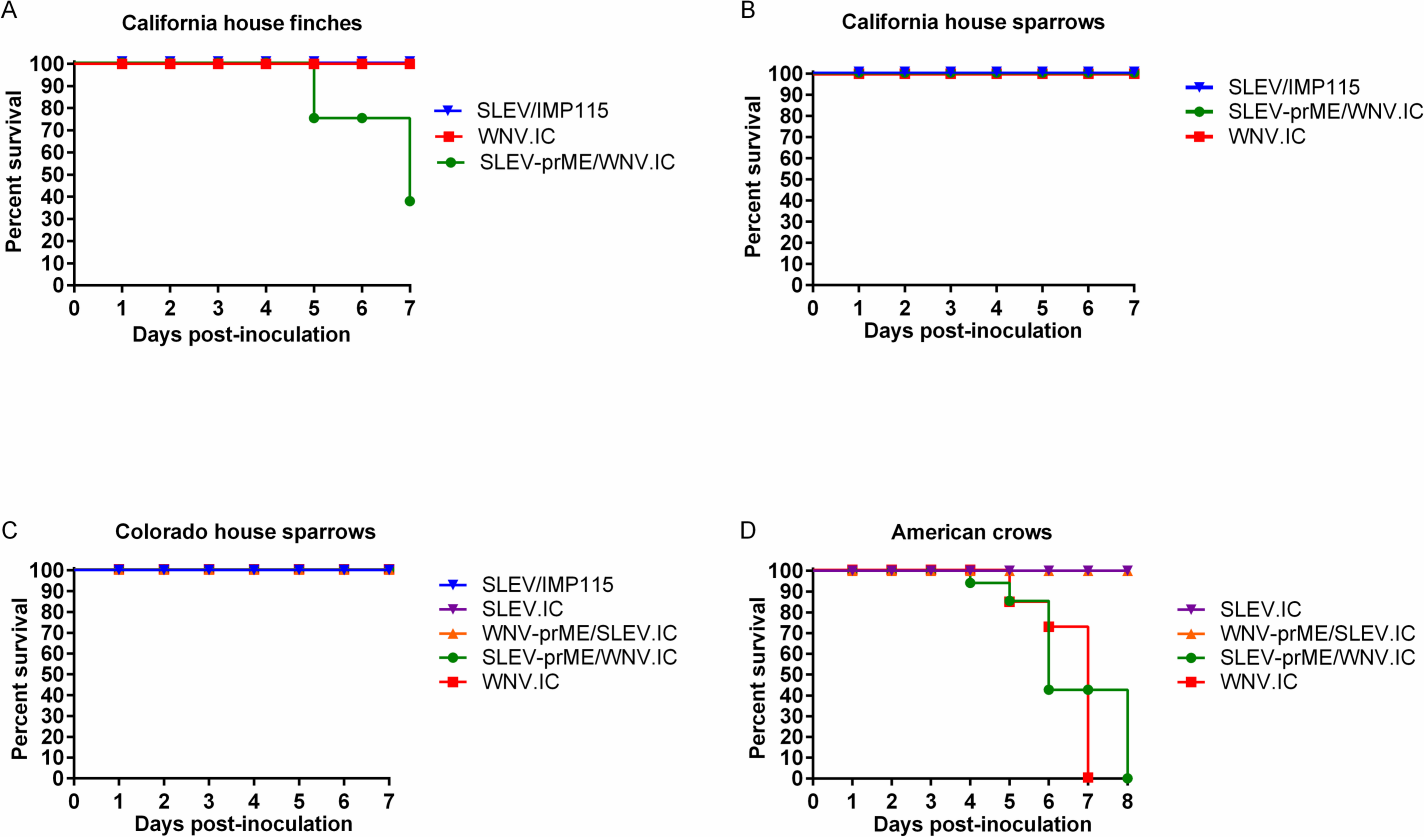
Survivorship curves (dpi 0–7) for (A) California house finches (B) California house sparrows, (C) Colorado house sparrows and (D) American crows inoculated with SLEV.IC, SLEV/IMP115, WNV-prME/SLEV.IC, SLEV-prME/WNV.IC and WNV.IC viruses.

California HOFIs inoculated with SLEV/ IMP115 produced significantly lower (p<0.05) daily mean titers in comparison with SLEV-prME/WNV.IC and WNV.IC (Fig 1A). In contrast, growth of SLEV-prME/WNV.IC was statistically indistinguishable (p>0.05) from WNV.IC (Fig 1A). Peak mean titers showed a similar pattern where SLEV/IMP115 was 3.5 ± 1.0 log_10_ PFU/mL and significantly lower (p<0.05) than SLEV-prME/WNV.IC and WNV.IC titers (Fig 2A). Peak mean titers of SLEV-prME/WNV.IC (6.8 ± 0.6 log_10_ PFU/mL) and WNV.IC (7.4 ± 1.0 log_10_ PFU/mL) were statistically indistinguishable (p>0.05) (Fig 2A). No morbidity or mortality was observed with HOFIs inoculated with WNV.IC or SLEV/IMP115 (Fig 3A). In contrast, only two HOFIs died by 5 dpi and only one HOFI survived in the SLEV-prME/WNV.IC group by 7dpi (Fig 3A).

California HOSPs inoculated with the SLEV/IMP115 parental virus produced daily mean titers that were approximately 10,000-fold and 100-fold lower (p<0.05) than WNV.IC and SLEV-prME/WNV.IC, respectively at 3–4 dpi (Fig 1B). Daily mean titers for SLEV-prME/WNV.IC were significantly lower than WNV.IC titers by 100-fold (p<0.05) and significantly higher than SLEV-IMP115 titers (p<0.05) by approximately 100-fold at 3–4 dpi (Fig 1B). The peak mean titer of SLEV/IMP115 was 3.1 ± 1.3 log_10_ PFU/mL, WNV-prME/SLEV.IC was 5.3 ± 1.0 log_10_ PFU/mL and WNV.IC-inoculated HOSPs was 7.3 ± 1.4 log_10_ PFU/mL (Fig 2B); peak mean titers from individual birds for all three viruses were significantly different (p<0.05) from each other (Fig 2B). No mortality was observed in any CA HOSP group (Fig 3B).

The viremia profiles of CO HOSPs inoculated with WNV.IC and SLEV-prME/WNV.IC were indistinguishable (p>0.05), but were higher (p<0.05) than those of SLEV/IMP115, SLEV.IC and WNV-prME/SLEV.IC during 2–4 dpi (Fig 1C). The viremia profiles for SLEV/IMP115, SLEV.IC and WNV-prME/SLEV.IC were statistically indistinguishable (p>0.05) and peaked on 6 dpi (Fig 1C), 2–3 dpi later than SLEV/IMP115 in California HOSPs (Fig 1B). House sparrows inoculated with WNV.IC and SLEV-prME/WNV.IC produced indistinguishable (p>0.05) peak mean titers of 5.9 ± 1.0 log_10_ PFU/mL and 6.0 ± 1.8 log_10_ PFU/mL, respectively (Fig 2C). In contrast, SLEV/IMP115, SLEV.IC and WNV-prME/SLEV.IC produced lower (p<0.05) peak mean titers of 4.4 ± 1.3 log_10_ PFU/mL, 4.4 ± 1.0 log_10_ PFU/mL and 3.0 ±1.5 log_10_ PFU/mL, respectively (Fig 2C). Mean peak titers for this latter group were not significantly different (p<0.05). No mortality was observed in these CO HOSPs after primary infection (Fig 3C).

American crows inoculated with WNV.IC and SLEV-prME/WNV.IC demonstrated markedly higher viremias (Fig 1D) than SLEV.IC and WNV-prME/SLEV.IC, with statistically indistinguishable peak mean titers of 11.9 ± 0.7 and 11.9 ± 0.3 log_10_ PFU/mL sera, respectively (Fig 2D). In contrast, AMCRs inoculated with SLEV.IC and WNV-prME/SLEV. IC failed to generate viremias above the limit of detection of 1.7 log_10_ PFU/mL sera. Peak mean titers for SLEV-prME/WNV.IC and WNV.IC were 10 billion times higher (p<0.0001) than SLEV.IC and WNV-prME/SLEV.IC (Fig 2D). American crows inoculated with WNV.IC and SLEV-prME/WNV.IC demonstrated 100% mortality by 7–8 dpi, while 100% survivorship was observed in AMCRs inoculated with the SLEV.IC and WNV-prME/SLEV.IC viruses (Fig 3D).

### WNV/SLEV prME-mediated cross-immunity

**Fig 4:**
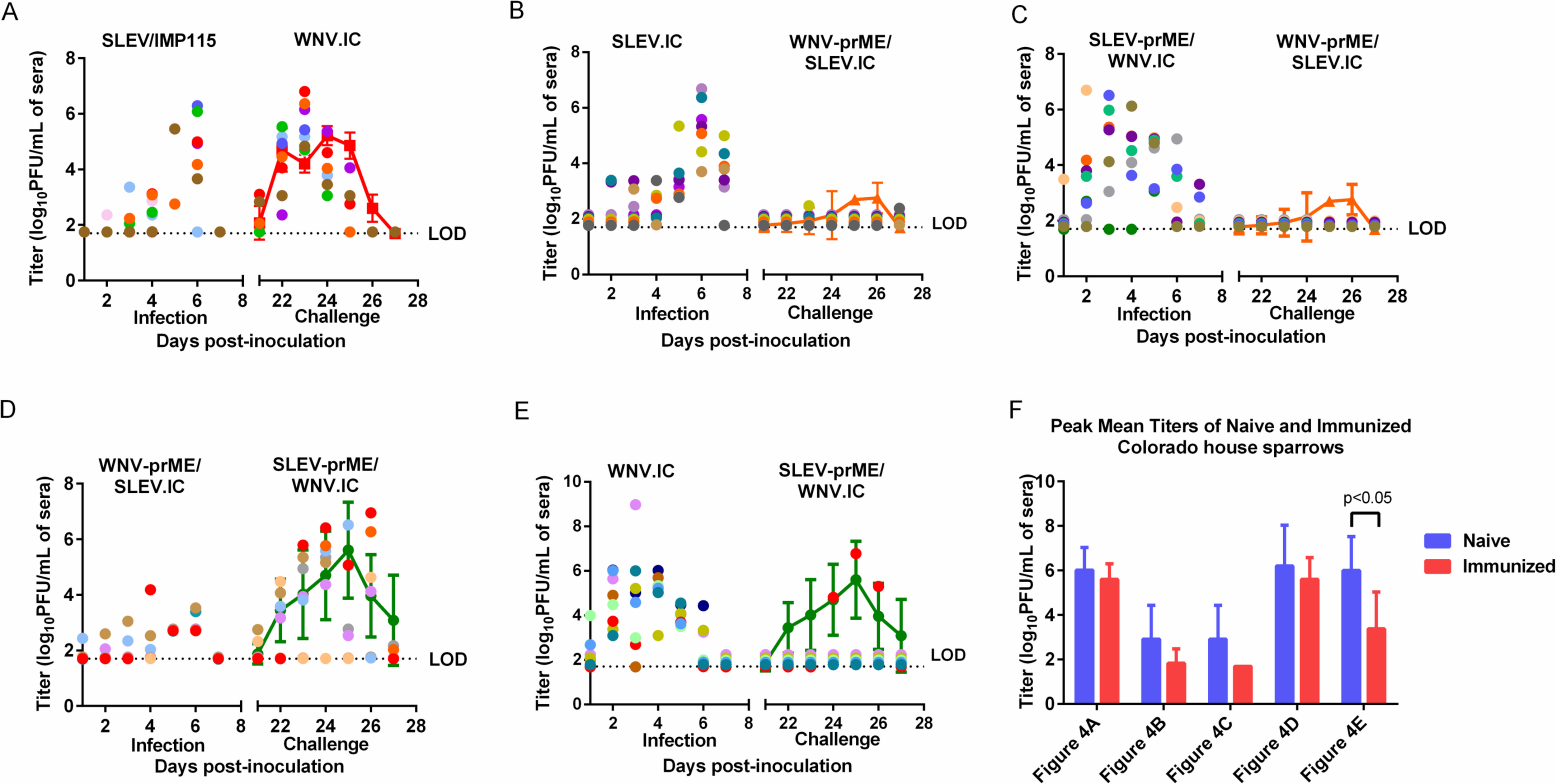
Daily serum virus titers in log_10_ PFU/mL were plotted over 7 days post-inoculation (dpi) for WNV/SLEV naïve and WNV/SLEV immune house sparrows (HOSP). Figures A-E depict HOSPs that were initially inoculated (scatter plots) with SLEV/IMP 115 (A), SLEV.IC (B), SLEV-prME/WNV.IC (C), WNV-prME/SLEV.IC (D) or WNV.IC (E) and then challenged with antigenically heterologous viruses, WNV.IC (A), WNV-prME/SLEV.IC (B, C) or SLEV-prME/WNV.IC (E, F) at 21 dpi. The data points on the scatter plots were off-set by a value of 0.01 for graphing purposes. For comparison purposes, line graphs representing daily mean virus titers (± 95% confidence intervals) of WNV.IC, WNV-prME/SLEV.IC or SLEV-prME/WNV.IC in naïve HOSPs were included. Titers from challenged HOSPs that fell outside of the 95% confidence limits of naïve HOSP titers were considered significantly different (p<0.05). (F) Peak mean virus titers for both naïve and previously inoculated HOSPs from Fig 4A–4E. The blue bars represent peak mean titers in naïve HOSPs inoculated with WNV.IC, WNV-prME/SLEV.IC or SLEV-prME/WNV.IC viruses. The red bars represent peak mean titers in immunized HOSPS challenged with WNV.IC, WNV-prME/SLEV.IC or SLEV-prME/WNV.IC viruses. The detection limit (LOD) for serum virus titers was 1.7 log_10_ PFU/mL.

Previously inoculated CO HOSPs were challenged at 21 dpi with antigenically heterologous viruses, including WNV.IC, WNV-prME/SLEV.IC or SLEV-prME/WNV.IC (Table 2). Viremias were assessed from 21–27 dpi with the secondary challenge virus (Fig 4).

One CA HOFI that seroconverted to WNV after antibody screening was inoculated with SLEV-prME/WNV.IC (S1 Fig). Virus titers in this bird were significantly lower (p<0.05) at 2–3 dpi in comparison with naïve birds inoculated with SLEV-prME/WNV.IC (S1 Fig). At 4 dpi, SLEV-prME/WNV.IC titer in the WNV-seropositive HOFI reached 7.0 log_10_ PFU/mL which was higher than the daily mean virus titer of 5.1 log_10_ PFU/mL in naïve HOFIs but was not outside the 95% confidence interval of ± 2.1 log_10_ PFU/mL. At dpi 5, the SLEV-prME/WNV.IC titer of the WNV-seropositive HOFI was significantly higher (p<0.05) than daily mean titer of SLEV-prME/WNV.IC in naïve HOFIs (S1 Fig). The WNV-immune HOFI was euthanized on 6 dpi. No other significant differences were detected at any other time point.

In SLEV/ IMP115-immune HOSPs, daily individual titers from secondary WNV.IC inoculations were lower than the 95% CIs of WNV.IC titers in naïve HOSPs at 24 and 25 dpi (Fig 4B). At 21 dpi, 2 HOSPs had significantly higher titers than WNV.IC titers in naïve HOSPs. Notably, at 23 dpi, all SLEV/ IMP115-immune HOSPs had WNV.IC titers that fell outside the upper 95% CI of the daily mean WNV.IC titer in naïve HOSPs. There were no significant differences (p>0.05) in peak mean titers for WNV.IC between naïve and challenged HOSPs (Fig 4F). One SLEV/ IMP115-immune HOSP succumbed to infection on 26 dpi following WNV challenge.

Daily individual viral titers of WNV-prME/SLEV.IC in SLEV.IC-immune HOSPs were either undetectable or lower than daily mean titers from WNV-prME/SLEV.IC inoculated naïve HOSPs (Fig 4C). WNV-prME/SLEV.IC viremias were detectable in only 2 of the 8 SLEV.IC-immune HOSPs at 23 dpi, with a peak mean titer of 2.2 ± 0.1 log_10_ PFU/mL (Fig 4F). No mortality was observed in the challenged HOSPs. There was no detectable viremia in any SLEV-prME/WNV.IC-immune HOSP challenged with the WNV-prME/SLEV.IC virus (Fig 4D). In comparison, naïve HOSPs inoculated with WNV-prME/SLEV.IC demonstrated a significantly higher (p<0.05) peak mean titer of 3.0 ± 1.8 log_10_ PFU/mL (Fig 2C).

WNV-prME/SLEV.IC- immune HOSPs challenged with the reciprocal chimeric virus, SLEV-prME/WNV.IC produced daily mean titers that were not significantly different (p>0.05) from daily mean titers in naive HOSPs inoculated with SLEV-prME/WNV.IC (Fig 4E). Peak mean titers between naïve and challenged HOSPs were not significantly different (p>0.05) (Fig 4F). However, one HOSP (red scatter plot) developed SLEV-prME/WNV.IC titers that were significantly higher (p<0.05) than naïve HOSPs at 23, 24 and 26 dpi. Another WNV-prME/SLEV.IC- immune HOSP (orange scatter plot) also produced statistically higher SLEV-prME/WNV.IC titers at 26 dpi (p<0.05).

Only one WNV.IC-immune HOSP developed a detectable viremia after challenge with SLEV-prME/WNV.IC (Fig 4F) with peak titer of 6.8 log_10_ PFU/mL by 25 dpi (Fig 4F); these titers were not statistically significant different from daily mean SLEV-prME/WNV.IC titers in naïve HOSPs. Viremia was undetectable in all other HOSPs. In comparison, primary infection with SLEV-prME/WNV.IC in naïve HOSPs elicited a mean peak titer of 6.0 ± 1.5 log_10_ PFU/mL, which was significantly higher (p<0.05) than the mean peak titer of 3.37 ± 1.7 log_10_ PFU/mL in the challenged HOSPs (Fig 4F). There was 33% mortality rate by 25 dpi.

Naïve HOSPs inoculated with the SLEV-prME group of viruses, i.e. SLEV/IMP115 (S2A Fig), SLEV.IC (S2B Fig) and SLEV-prME/WNV.IC (S2C Fig), produced SLEV-prME neutralizing antibodies (SLEV-prME/WNV.IC naïve and challenge) of variable titers that ranged from 1:20–1:160. Protective cross-neutralizing WNV-prME antibodies were not detected with these HOSPs. Naïve HOSPs inoculated with either WNV-prME/SLEV.IC (S2D Fig) or WNV.IC (S2E Fig) produced a variable range of both SLEV-prME and WNV-prME neutralizing antibody titers that were between 16 to 128-fold higher than the neutralizing antibody titers from SLEV-prME inoculated HOSPs.

House sparrows that were SLEV-prME immune and then challenged with heterologous WNV-prME viruses; WNV-prME/SLEV.IC or WNV.IC respectively (S2A–S2C Fig) produced 16 to 64-fold higher SLEV-prME neutralizing antibodies when compared with naïve HOSPs. WNV-prME neutralizing antibody titers also increased 20 to 2,560-fold in the SLEV-prME immune HOSPs in comparison with naïve HOSPs. The 2 groups of SLEV-prME immune HOSPs that were subsequently challenged with WNV-prME/SLEV.IC produced at least 16- fold higher WNV-prME neutralizing antibody titers after challenge (S2B and S2C Fig) than SLEV-prME immune HOSPs challenged with just WNV.IC (S2A Fig). Interestingly, there was no detectable WNV-prME/SLEV.IC viremia production after challenge, with the exception of 2 HOSPs (barely above the detection limit of 1.7 log_10_PFU/mL) in these two groups (Fig 4C and 4D). Whereas in SLEV-prME immune HOSPs, WNV.IC was able to replicate to similar titers as that observed in naïve HOSPs (Fig 4B).

By 21 dpi post-challenge, only 38% of the HOSPs survived in the WNV-prME immune groups that were challenged with antigenically heterologous SLEV-prME/WNV.IC. Neutralizing and cross-neutralizing antibody titers against both WNV-prME and SLEV-prME antigens in these remaining HOSPs were either 20 or below detection (S2 Fig). In the WNV-prME/SLEV.IC immune group, all HOSPs were able to produce SLEV-prME/WNV.IC titers when challenged (Fig 4E). In subsequent challenge with SLEV-prME/WNV.IC, WNV.IC-immune HOSPs did not produce detectable viremias with the exception of one HOSP (Fig 4F).

## Discussion

Virulence differences between WNV and SLEV in avian hosts have been well documented [7, 16, 34–38]. Compared to SLEV, WNV elicits significantly higher viremias in a wide range of avian hosts [7, 15, 17, 34, 35, 39, 40]. Data from the present study further support these avian host competence associations, where WNV elicited significantly higher viremias than SLEV in HOSPs, HOFIs and AMCRs. Inoculations of AMCRs further demonstrated that certain avian hosts are extremely competent for WNV replication, but fail to generate a detectable SLEV viremia [6]. Although corvids likely amplify WNV to levels leading to outbreaks of human disease [41], they conversely may suppress SLEV activity by serving as dead end hosts for this virus.

Specific viral genetic differences have not been implicated in the explanation of the disparate phenotypes displayed by WNV and SLEV in birds. Data presented here clearly demonstrate that the non-structural (NS) genetic elements of WNV confer an elevated avian host competence phenotype in three different Passeriform hosts, HOFIs, HOSPs and AMCRs. In species like the California HOFI, incorporation of WNV NS elements resulted in a significantly higher mortality rate as well. In the AMCR model, SLEV NS elements ablated virus replication completely clearly showing that competence of AMCRs for SLEV replication is dependent on SLEV NS elements. This extends previous *in vitro* findings where viral growth kinetics in DEF cells and cytopathogenicity profiles in Vero cells were found to be enhanced by WNV NS viral genetic elements [27]. Studies investigating the specific genetic determinants of differential avian virulence between different WNV strains previously have been assessed. Notably, altering the amino acid identity at the 249 position in the helicase domain of the NS3 protein from a threonine to a proline (NS3-T249P) conferred elevated viremias and subsequent virulence modulation in AMCRs and HOSPs [2, 25, 42]. Polymorphisms at this loci also were associated with modulated *ex vivo* AMCR PBMC growth competence that were associated with *in vivo* AMCR virulence phenotypes [43]. Modification of this locus with the SLEV backbone failed to generate viable virus (Dietrich EA, personal communication). However, the results from *ex vivo* WNV studies indicated that peripheral leukocytes were likely a key cell population for high viremia production, and differential intracellular growth potential modulated by the NS proteins of WNV and SLEV could be a factor limiting peripheral viremias elicited in SLEV infected avian hosts. NS1’, a translational extension of the native NS1 due to ribosomal frame-shifting, has been implicated as a determinant of elevated viremia production in HOSPs in addition to a requisite for murine neuroinvasiveness [44, 45]. Production of the NS1’ has been implicated with the maintenance of a specific stoichiometric relationship between structural and nonstructural elements leading to an overexpression of structural proteins [45]. Mutant WNV viruses that were defective in producing the NS1’ protein have been shown to exhibit decreased viral titers in HOSPs in comparison with wild-type WNV [45]. As the NS1’ protein has not been predicted to be formed by SLEV due to the lack of a heptanucleotide slippery sequence motif that is a requisite for ribosomal frame-shifting [44]; the absence of this protein or the lack of stoichiometric imbalance that would be predicted to result from SLEV replication could have contributed to the overall lower levels of SLEV replication observed both *in vitro* and *in vivo*.

In this study, a WNV field-immune CA HOFI was inadvertently inoculated with SLEV-prME/WNV.IC. The SLEV-prME/WNV.IC titers in the WNV-immunized HOFI were significantly lower than titers in naïve HOFIs at 2–3 dpi and reached a peak titer at 4dpi. In contrast peak SLEV-prME/WNV.IC titer in naïve HOFIs was reached by 2 dpi. The delay in reaching a peak titer and the lower titers at the earlier timepoints for the WNV-immunized HOFI suggests that WNV cross-neutralizing antibodies that form after two weeks of infection were not able to completely ablate subsequent infection by an immunologically heterologous chimeric flavivirus such SLEV-prME/WNV.IC. In contrast, Fang et al. demonstrated that when WNV-immune HOFIs were challenged with SLEV at 6 weeks post initial infection, the WNV-immunized HOFIs were completely protected from SLEV infection [16]. Thus suggesting that the level of cross-neutralization between WNV and SLEV could depend on the time difference between primary and secondary heterologous/ homologous infections.

With the Colorado HOSP challenge study, a similar trend was observed with WNV-immune HOSPs that were challenged with SLEV-prME/WNV.IC. In this group, only one HOSP developed a viremia with SLEV-prME/WNV.IC that was similar to that observed in naive HOSPs. No viremia was detected in any other HOSP. Higher SLEV-prME/WNV.IC titers were observed at 25–26 dpi in the WNV-immune HOSP in comparison with naïve HOSPs; however, statistical significance was not achieved due to the variable SLEV-prME/WNV.IC viremia levels in these wild-caught naïve HOSPs. With WNV-prME/SLEV.IC-immune HOSPs challenged with antigenically heterologous SLEV-prME/WNV.IC, one WNV-prME/SLEV.IC-immune HOSP consistently produced significantly higher SLEV-prME/WNV.IC titers than daily mean SLEV-prME/WNV.IC titers in naïve HOSPs from 23–26 dpi. A second WNV-prME/SLEV.IC-immune HOSP also generated higher SLEV-prME/WNV.IC titers than naïve HOSPs on 26 dpi. Several SLEV-immune HOSPs produced significantly higher WNV titers than titers observed in naïve HOSPs but this was only observed at 23 dpi. These data demonstrated the potential generation of SLEV and WNV viremias in the presence of heterologous immunity and statistically higher titers in some previously infected birds than in naïve birds. Taken together this suggests that pre-existing WNV/SLEV immunity in passerines does not preclude replication of antigenically heterologous viruses. It also suggests that WNV/SLEV could replicate to titers high enough in previously infected passerines to meet the oral infection thresholds of *Culex* spp. mosquitoes [46] thus contributing to the WNV/SLEV natural transmission cycle.

Similar to previous studies in HOFIs [18], prior immunity to SLEV did not preclude viremia generation following WNV infection in HOSPs. These observations coupled with the additional findings that WNV mediated elevated avian viremias due to NS genetic elements provided an elemental foundation to explain the capacity of WNV to emerge in North America in geographic regions where avian SLEV immunity pre-existed. The experimental model demonstrated here in which a virus has been artificially generated with the antigenic properties of SLEV and the avian viremia potential of WNV (SLEV-prME/WNV) demonstrated that unlike previous experiments in which HOFIs with pre-existing immunity to WNV failed to develop viremias following subsequent SLEV inoculation, an SLEV antigenic virus capable of replicating to higher titers in birds overcame cross neutralization and produced titers potentially infectious to mosquitoes. The 2015 SLEV outbreak in Arizona could have resulted from the emergence of a novel SLEV genotype with increased avian viremia potential or a variant that could have utilized cross-reactive WNV immunoglobulin to enhance infectivity of PBMCs or from an SLEV strain that had incorporated genetic changes reducing cross-neutralizing determinants. Unlike the lineage V SLEV strain utilized in these studies, the Arizona SLEV outbreak resulted from the introduction of a novel genotype III SLEV strain [19]. This recent evidence of WNV and SLEV co-circulation in Arizona and California indicate that the competitive exclusion of SLEV by WNV is not complete and will require a re-evaluation with additional strains of both viruses and their subsequent cross neutralization potential within avian hosts.

Host competence incorporates a combination of three factors: susceptibility to infection, mean daily infectiousness (estimated number of vectors that could become infected per day) and duration of viremia [47, 48]. Based on the host competence index calculation, for which a uniform vector competence phenotype for both viruses would be modeled, WNV was predicted to have increased infectiousness for mosquitoes compared to SLEV. However, SLEV has been shown to exhibit a significantly lower mosquito oral infection threshold compared to WNV [34, 46]. This suggests that SLEV could compensate for relatively low host competence in birds with high oral infectivity for *Culex* mosquito vectors. Under this scenario, SLEV would have adapted to specific North American mosquito vectors such that transmission could occur in the absence of elevated avian viremias that lead to high host mortality. This assertion is supported by SLEV having experienced a considerably longer period of adaptation to New World vectors than WNV, with adaptive radiation of SLEV predicted from phylogenetic reconstructions to have occurred >300 years ago [49]. The potential for WNV to undergo similar adaptation to North American vectors could be limited by fitness trade-offs in avian hosts that could be exacerbated by the fact that both vector and host competency for WNV and SLEV are mediated by NS genetic elements [27, 46].

## Supporting information

S1 Fig Graph depicts the daily viremia profile of a field-collected WNV-seropositive California HOFI that was inadvertently challenged with SLEV-prME/WNV.IC (n = 1; black) in comparison with the daily mean serum titers of naïve CA HOFIs inoculated with SLEV-prME/WNV.IC (n = 4; green line line graph). The detection limit (LOD) for serum virus titers was 1.7 log_10_ PFU/mL.
(TIF)

S2 Fig PRNT_90_ titers for WNV/SLEV immune house sparrows inoculated with heterologous viruses (Table 2). Neutralization of both WNV.IC and SLEV-prME/WNV.IC was tested using a standard PRNT_90_ assay. Graphs show PRNT_90_ titers for WNV.IC and SLEV-prME/WNV.IC in naïve and challenged HOSPs: (A) SLEV/IMP115-immune HOSPs challenged with WNV.IC, (B) SLEV.IC-immunized HOSPs challenged with WNV-prME/SLEV.IC virus, (C) SLEV-prME/WNV.IC-immune HOSPs challenged with WNV-prME/SLEV.IC, (D) WNV-prME/SLEV.IC immune HOSPs challenged with SLEV-prME/WNV.IC (E) WNV.IC immune HOSPs challenged with SLEV-prME/WNV.IC virus.
(TIF)
