# Supplementary figures and images for: West Nile and St. Louis encephalitis viral genetic determinants of avian host competence
Source: PLoS Negl Trop Dis. 2018 Feb 15;12(2):e0006302. doi: 10.1371/journal.pntd.0006302 (PMC5831645; doi:10.1371/journal.pntd.0006302)

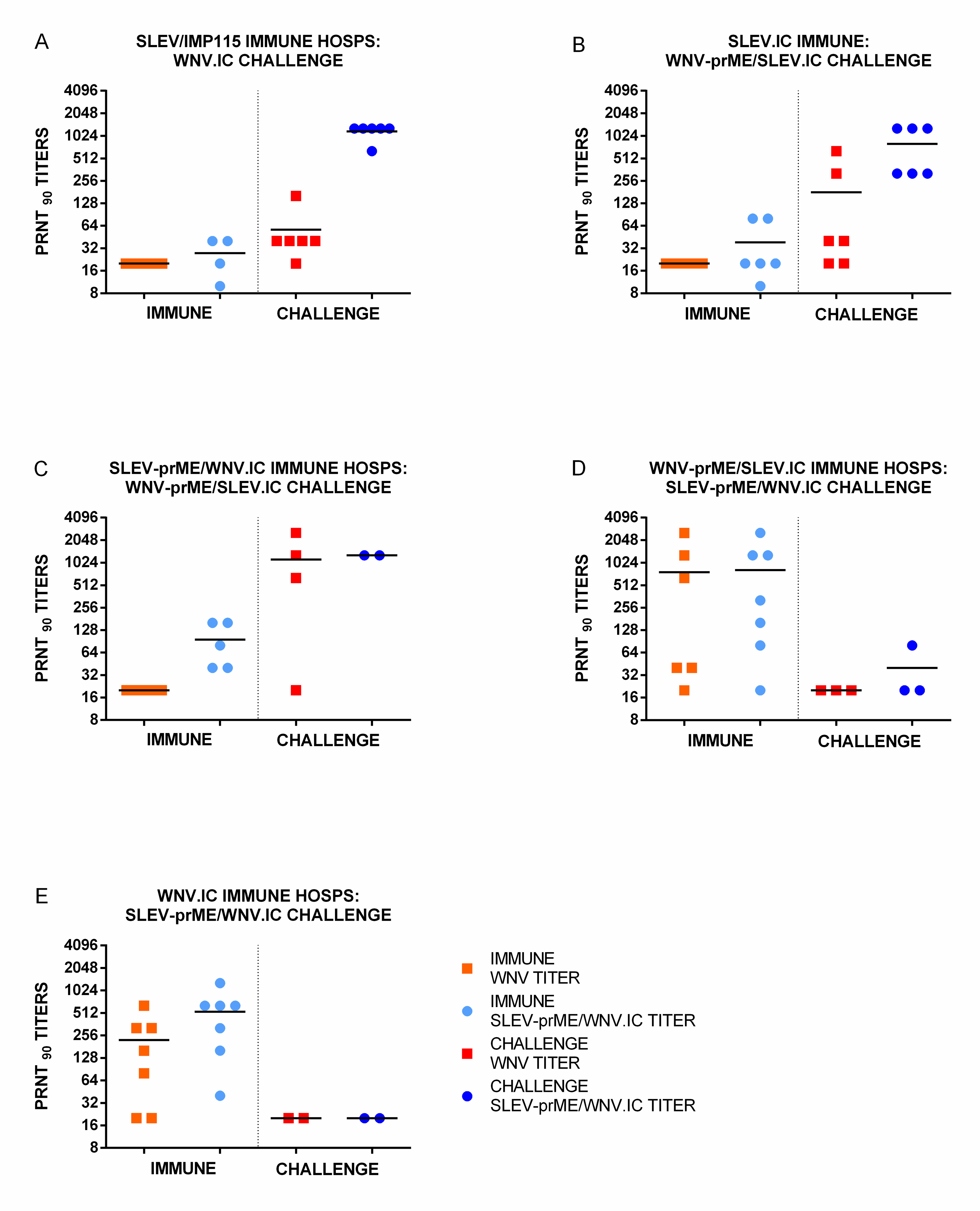

Supplement: S2 Fig — Neutralization of both WNV.IC and SLEV-prME/WNV.IC was tested using a standard PRNT90 assay. Graphs show PRNT90 titers for WNV.IC and SLEV-prME/WNV.IC in naïve and challenged HOSPs: (A) SLEV/IMP115-immune HOSPs challenged with WNV.IC, (B) SLEV.IC-immunized HOSPs challenged with WNV-prME/SLEV.IC virus, (C) SLEV-prME/WNV.IC-immune HOSPs challenged with WNV-prME/SLEV.IC, (D) WNV-prME/SLEV.IC immune HOSPs challenged with SLEV-prME/WNV.IC (E) WNV.IC immune HOSPs challenged with SLEV-prME/WNV.IC virus. (TIF) [file pntd.0006302.s002.tif]
